# Future Perspectives of POSEIDON Stratification for Clinical Practice and Research

**DOI:** 10.3389/fendo.2019.00439

**Published:** 2019-07-11

**Authors:** Peter Humaidan, Antonio La Marca, Carlo Alviggi, Sandro C. Esteves, Thor Haahr

**Affiliations:** ^1^Department of Clinical Medicine, Aarhus University, Aarhus, Denmark; ^2^Fertility Clinic Skive, Skive Regional Hospital, Skive, Denmark; ^3^Department of Obstetrics, Gynecology and Pediatric Sciences, Institute of Obstetrics and Gynecology, University of Modena, Modena, Italy; ^4^Department of Neuroscience, Reproductive Science, and Odontostomatology, University of Naples, Federico II, Naples, Italy; ^5^Istituto per l'Endocrinologia e l'Oncologia Sperimentale, Consiglio Nazionale Delle Ricerche, Naples, Italy; ^6^ANDROFERT, Andrology and Human Reproduction Clinic, Campinas, Brazil; ^7^Department of Surgery, University of Campinas, Campinas, Brazil

**Keywords:** ART calculator, Bologna criteria, blastocyst, controlled ovarian stimulation, low ovarian response, pregnancy, POSEIDON criteria

## Abstract

A total of 50% of patients undergoing IVF treatment has previously been estimated to fulfill the POSEIDON classification criteria; importantly, although the reproductive prognosis differs between patients, POSEIDON patients share the same characteristic of a low ovarian response to exogenous gonadotropin stimulation—independent of age. POSEIDON patients require focused attention as regards ovarian stimulation in order to increase the chances of having at least one euploid blastocyst for transfer—the success criterion for stimulation set forth by the POSEIDON Group. The key to success seems to be individualization in all steps of treatment. In this perspective article we discuss the future impact of the POSEIDON stratification for daily clinical practice as well as for research.

## Future Perspectives of POSEIDON Stratification for Daily Clinical Practice

**Figure 1:**
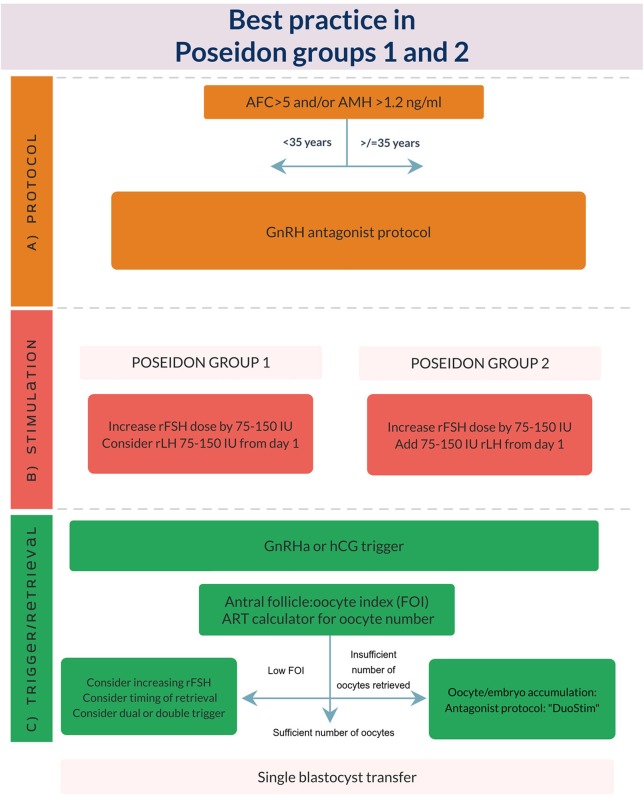
Best practice in POSEIDON groups 1 and 2. **(A)** POSEIDON recommends use of GnRH antagonist co-treatment for all patients in POSEIDON groups 1 or 2. **(B)** Ovarian stimulation strategy: First choice should be an increase in daily rFSH dose by 75–150 IU. In POSEIDON Group 1 patients with follicular stagnation at follicle sizes 1–12 mm, rLH 75–150 IU daily should be added from day 1 of stimulation. In POSEIDON 2 patients rLH 75–150 IU daily should be added to all patients from day 1 of stimulation. **(C)** Ovulation trigger strategy: GnRHa is mandatory in the follicular phase stimulation of the DuoStim protocol. All trigger agents can be used in the luteal phase stimulation. In non DuoStim GnRH antagonist cycles, the choice of trigger between GnRHa and hCG should rely on the embryo transfer strategy (fresh or frozen), patient characteristics (e.g., hypo-hypo) and clinical experience. In cases with a low FOI (4), clinicians should consider pretreatment including short term estrogen or progestin therapy, or OCP for synchronization of the follicles prior to stimulation, adjuvant LH activity during stimulation, or changing trigger strategy to either dual or double trigger. In case of an insufficient number of oocytes retrieved as determined by the ART calculator, the probability of transferring a euploid embryo should be discussed with the patient to counsel whether an immediate transfer or a new stimulation should be suggested (3).

**Figure 2:**
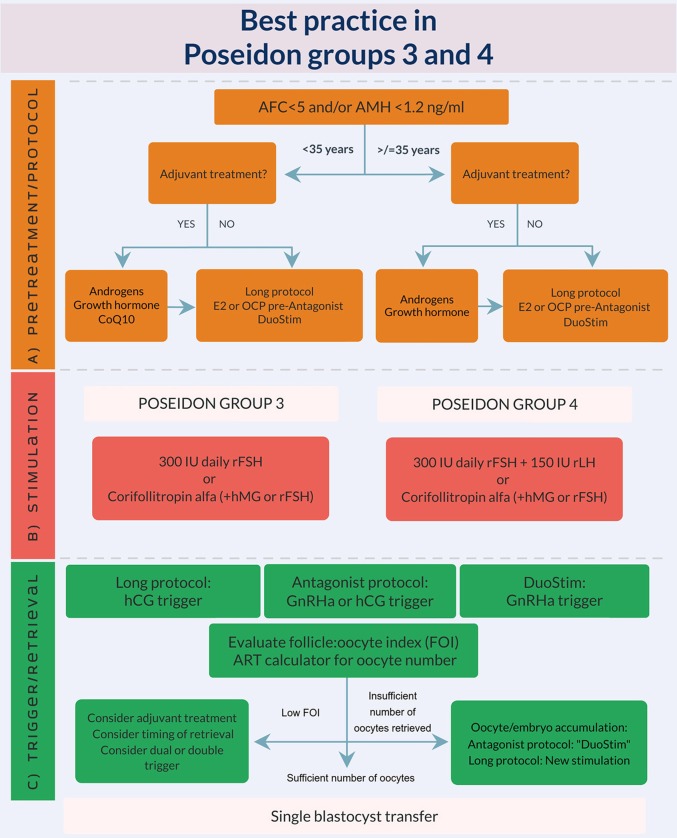
Best practice in POSEIDON groups 3 and 4. **(A)** Pretreatment is rarely the first option in low prognosis patients, but in case of low response to ovarian stimulation, e.g., asynchrony of follicular growth and inadequate ovarian response, pretreatment should be considered. The choice should rely on availability, clinical experience and patient preference. Stimulation protocol might start using GnRH antagonist co-treatment keeping in mind the possibility of converting to DuoStim to achieve the individualized oocyte number (according to the ART calculator). Otherwise a long GnRHa protocol should be considered first choice. **(B)** Ovarian stimulation strategy: First choice in Poseidon group 3 is the GnRH antagonist cycle using either 300 IU daily of rFSH alone or Corifollitropin alfa followed by either rFSH or hMG. In POSEIDON group 4 patients, rLH (75–150 IU daily) should be added from day 1 of stimulation, unless the combination of Corifollitropin alfa and hMG was chosen. GnRH antagonist co-treatment allows the use of Duostim. **(C)** Ovulation trigger strategy: In the long GnRHa down-regulation protocol hCG is mandatory as ovulation trigger, whereas GnRHa is mandatory in the follicular phase stimulation of the DuoStim protocol. All trigger agents can be used for the luteal phase stimulation. In non DuoStim GnRH antagonist cycles, the choice of trigger between GnRHa and hCG should rely on the embryo transfer strategy (fresh or frozen), patient characteristics, and clinical experience. In cases with a low antral follicle to oocyte ratio (FOI) as determined on trigger day (4), clinicians should consider: pretreatment including short term estrogen or progestin therapy, or OCP for synchronization of the follicles prior to stimulation, adjuvant LH activity during stimulation, or changing trigger strategy to either dual or double trigger. In case of an insufficient number of oocytes retrieved as determined by the ART calculator, the probability of transferring a euploid embryo should be discussed with the patient to counsel whether an immediate transfer or a new stimulation should be suggested (3).

As previously mentioned in this supplement, the incitement to propose the POSEIDON criteria was the high degree of heterogeneity seen in the ESHRE Bologna criteria population (1, 2). With the new POSEIDON stratification significantly more homogenous sub-populations were created, taking age, ovarian reserve, and previous ovarian responses after stimulation with gonadotropins into account. Thus, the overall idea of the POSEIDON stratification was to not only guide clinicians regarding clinical management of the patient, but also to be a counseling tool to help set patient expectations prior to initiation of ovarian stimulation. For this purpose, a number of clinical recommendations in terms of type of GnRH analog, gonadotropin type and dosing were suggested in order to obtain the new marker of success: the number of oocytes needed in each individual patient to obtain one euploid blastocyst. This quite naturally led to the subsequent development of the ART calculator (3). As seen from Figures 1, 2 the main factor distinguishing Groups 1 and 2 from Groups 3 and 4 is the ovarian reserve and a previous response to stimulation with exogenous gonadotropins (1, 2, 5).

Until now clinical management of the low prognosis patient has primarily been based on small studies including heterogenous populations which left clinicians with poor evidence to manage the low prognosis patient—and often a “trial and error” strategy was adopted by individual clinicians. With the POSEIDON stratification the clinician will very quickly get an impression of whether the individual patient fulfills the criteria of being a POSEIDON patient—and if positive (~50%)—to which of the four groups the patient belongs (6). This places POSEIDON as a daily partner in the clinic; moreover, the POSEIDON patient generally is a patient who needs more clinical consideration and individualization when compared to the other half of patients, constituted by non-POSEIDON patients.

As mentioned previously, patients in POSEIDON groups 1 and 2 underwent one or more stimulations leading to an unexpected impaired ovarian response. Either a low response resulting in <4 oocytes (Groups 1a and 2a) or a suboptimal response, resulting in 4–9 oocytes (Groups 1b and 2b). As seen from Figure 1 it is suggested that POSEIDON groups 1 and 2 patients undergo their next stimulation with an increase in rFSH dosing, rLH supplementation from day 1 of stimulation as well as GnRH antagonist co-treatment. The main difference between groups 1 and 2, is age and consequently, a difference in oocyte euploidy, and thus, reproductive potential. In general, one could classify the Group 1 patient as a patient with a good ovarian reserve—and due to her age also an expected good oocyte quality (7). In contrast, the Group 2 patient has an age-related increased oocyte aneuploidy although the ovarian reserve is good (7). This means that the number of oocytes needed to obtain success is significantly higher for the aging patient, but with her good ovarian reserve she is likely to reach the estimated number of oocytes needed for one euploid blastocyst (3). This means that future use of the POSEIDON stratification with or without the use of the ART calculator will help clinical decision-making as well as counseling.

Groups 3 and 4 are characterized by a low ovarian reserve which *per se* induces a poor reproductive prognosis. However, age makes a significant difference for success, and it is expected that the younger patient will have a four-times higher probability of a live birth per transfer as compared to the older patient– 20 vs. 5% (8). Again, the POSEIDON stratification will help clinical decision-making and counseling. As shown in Figure 2, the suggested handling of the POSEIDON group 3 patient would include either a long GnRHa down-regulation or a “primed” GnRH antagonist co-treatment (synchronization with short term estradiol or progestin treatment or oral contraceptive pill treatment) followed by stimulation with a maximum dose of 300 rFSH. In selected cases with a low oocyte yield and based on the estimate made by the ART calculator, DUO-Stim should be recommended for oocyte or embryo accumulation to shorten time to pregnancy (9–11).

With the increasing delay in child bearing, POSEIDON group 4 patients become more and more prevalent—in some centers constituting 55% of the POSEIDON population (6).The dual negative effect of a reduced ovarian reserve (quantity) as well as an age related increase in aneuploidy (quality) makes this category of patients difficult to handle (7). The POSEIDON recommendation for this patient would include either long GnRHa down-regulation or a “primed” GnRH antagonist co-treatment, followed by stimulation with a maximum dose of 300 rFSH and 150 IU rLH from day one of stimulation. In selected cases with low oocyte yield, DUO-Stim should be recommended for oocyte or embryo accumulation bearing in mind cost-efficiency—especially in women >40 years old (9–11). Although the initial attitude toward oocyte donation could be negative in a large proportion of older Group 4 patients (12), from a scientific point of view the best chance for a live birth would be oocyte donation.

Taken together, we see the POSEIDON stratification as a daily tool in future clinical practice, supporting not only clinical, but also patient decision-making.

## Future Perspectives of POSEIDON Stratification for Research

Currently, retrospective analyses of large databases may match patients so as to fit into one of the four POSEIDON groups. However, it is quite unlikely that patients were treated according to the recommendations made by the POSEIDON stratification. Thus, future RCT's are necessary to evaluate the stratification and the recommendations set forth in this supplement. In this aspect, POSEIDON groups 1 and 2 need to be studied separately from groups 3 and 4.

### Future Research in Groups 1 and 2

Groups 1 and 2 encompass good reserve patients, some of whom have the presence of FSH-R and LH-R polymorphisms or variant LHβ (13). Previous reports show that an increase in rFSH in patients with an unexpected low response to ovarian stimulation in the first stimulation cycle increases the number of oocytes retrieved which could be the effect of FSH receptor polymorphisms (14, 15). As regards rLH supplementation this has previously been proven to significantly increase clinical pregnancy rates (16–19). Future studies should evaluate the benefit of screening patients prior to their future stimulation for FSH-R and LH-R polymorphisms as well as variant LHβ (20). From the findings, the subsequent stimulation should be tailored accordingly; thus, patients with FSH-R polymorphisms should have an increase in FSH dosing, whereas patients with LH-R polymorphisms and presence of variant LHβ should be treated with rLH from day 1 of stimulation. The primary end point of these studies should be cumulative live birth (CLBR), i.e., the live births obtained after one embryo transfer and the subsequent frozen cycles within a 2–3-years period. The suggested secondary endpoint is the achievement of the number of mature oocytes needed to obtain at least one euploid blastocyst as per the ART calculator estimation (3).

### Future Research in Groups 3 and 4

The question asked for groups 3 and 4 is—can we increase the number of growing follicles and subsequently the number of competent oocytes? First of all, which GnRH analog regimen is the most optimal for Groups 3 and 4: the long GnRHa down-regulation protocol—or the GnRH antagonist protocol primed with either daily estradiol for 5 days prior to the unset of menses, or 12–14 days of oral contraceptive pills. Moreover, will long term pretreatment with androgens, or short-term pretreatment with growth hormone before and during stimulation have an effect on the number of growing follicles and oocytes? This question needs to be explored in future RCT's. Another pending question is whether DUOstim reduces time to live birth for groups 3 and 4. An RCT comparing DUOStim to a long GnRHa down-regulation protocol or a “primed” GnRH antagonist protocol is necessary to answer this question. Here again, cumulative live birth (CLBR), i.e., the live births obtained after one embryo transfer and the subsequent frozen cycles within a 2–3-years period will be the primary endpoint whereas the POSEIDON marker of success in ART, namely, the number of mature oocytes needed to obtain at least one euploid blastocyst as per the ART calculator estimation will be the secondary endpoint (3).

## Conclusions

The POSEIDON stratification has been well-accepted by reproductive endocrinologists and infertility specialists worldwide, however, this novel classification system needs to be prospectively investigated. It is our hope that during the following years POSEIDON and the ART calculator will be an integral part of daily clinical practice used for decision-making and counseling with the aims of providing the most optimal treatment of the patient and reducing time to live birth.

## Author Contributions

The style and concept were developed by PH and TH. All authors actively contributed to writing the manuscript and accepted the final version of this manuscript.

### Conflict of Interest Statement

TH received honoraria for lectures from Merck and Ferring. PH received unrestricted research grants from MSD, Merck, and Ferring as well as honoraria for lectures from MSD, Merck, Gedeon-Richter, Theramex, and IBSA. SE received honoraria for lectures from Merck, Lilly, Gedeon-Richter, and Besins. PH, CA, and SE are cofounders of the POSEIDON criteria. The remaining author declares that the research was conducted in the absence of any commercial or financial relationships that could be construed as a potential conflict of interest.
